# Radiomics-based prediction of multiple gene alteration incorporating mutual genetic information in glioblastoma and grade 4 astrocytoma, IDH-mutant

**DOI:** 10.1007/s11060-021-03870-z

**Published:** 2021-10-14

**Authors:** Beomseok Sohn, Chansik An, Dain Kim, Sung Soo Ahn, Kyunghwa Han, Se Hoon Kim, Seok-Gu Kang, Jong Hee Chang, Seung-Koo Lee

**Affiliations:** 1 Department of Radiology, Severance Hospital, Research Institute of Radiological Science and Center for Clinical Image Data Science, Yonsei University College of Medicine, Seoul, South Korea; 2 Department of Radiology and Research Institute, National Health Insurance Service Ilsan Hospital, Goyang, South Korea; 3 Department of Pathology, Yonsei University College of Medicine, Seoul, South Korea; 4 Department of Neurosurgery, Yonsei University College of Medicine, Seoul, South Korea

**Keywords:** Glioblastoma, Magnetic resonance imaging, Genes, Mutation, Brain

## Abstract

**Purpose:**

In glioma, molecular alterations are closely associated with disease prognosis. This study aimed to develop a radiomics-based multiple gene prediction model incorporating mutual information of each genetic alteration in glioblastoma and grade 4 astrocytoma, IDH-mutant.

**Methods:**

From December 2014 through January 2020, we enrolled 418 patients with pathologically confirmed glioblastoma (based on the 2016 WHO classification). All selected patients had preoperative MRI and isocitrate dehydrogenase (IDH) mutation, O-6-methylguanine-DNA methyltransferase (MGMT) promoter methylation, epidermal growth factor receptor amplification, and alpha-thalassemia/mental retardation syndrome X-linked (ATRX) loss status. Patients were randomly split into training and test sets (7:3 ratio). Enhancing tumor and peritumoral T2-hyperintensity were auto-segmented, and 660 radiomics features were extracted. We built binary relevance (BR) and ensemble classifier chain (ECC) models for multi-label classification and compared their performance. In the classifier chain, we calculated the mean absolute Shapley value of input features.

**Results:**

The micro-averaged area under the curves (AUCs) for the test set were 0.804 and 0.842 in BR and ECC models, respectively. IDH mutation status was predicted with the highest AUCs of 0.964 (BR) and 0.967 (ECC). The ECC model showed higher AUCs than the BR model for ATRX (0.822 vs. 0.775) and MGMT promoter methylation (0.761 vs. 0.653) predictions. The mean absolute Shapley values suggested that predicted outcomes from the prior classifiers were important for better subsequent predictions along the classifier chains.

**Conclusion:**

We built a radiomics-based multiple gene prediction chained model that incorporates mutual information of each genetic alteration in glioblastoma and grade 4 astrocytoma, IDH-mutant and performs better than a simple bundle of binary classifiers using prior classifiers’ prediction probability.

**Supplementary Information:**

The online version contains supplementary material available at 10.1007/s11060-021-03870-z.

## Introduction

Glioblastoma, the most common primary malignant brain parenchymal tumor, is challenging to treat [1]. A comprehensive molecular characterization of glioblastoma showed that most tumors harbor recurrent molecular alterations disrupting core pathways involved in the regulation of growth, cell cycle, DNA repair, apoptosis, and control of chromatin state [2]. These recurrent and relevant genomic variants continue to be targets for drug development [3–5].

Isocitrate dehydrogenase (IDH) mutation has been recognized as one of the most important molecular markers in gliomas and integrated for glioma classification since 2016 [6–8]. In addition, according to the upcoming 2021 WHO Classification of Tumors of the Central Nervous System (CNS) [9], previously called glioblastoma, isocitrate dehydrogenase (IDH)-mutant is now designated as *astrocytoma, IDH-mutant, WHO grade 4* and glioblastoma should be diagnosed in the setting of IDH wildtype. O-6-methylguanine-DNA methyltransferase (MGMT) promoter methylation and epidermal growth factor receptor (EGFR) alteration are also related to prognosis. Despite the potential benefits of these genetic biomarkers, methods such as next-generation sequencing still have limited clinical utility due to costs and the need for direct tissue sampling. Therefore, it is desirable that MRI predicts the tumors with specific molecular features. Several studies have investigated that quantitative image features from preoperative imaging of gliomas can be used to predict IDH and alpha-thalassemia/mental retardation syndrome X-linked (ATRX) mutations, EGFR amplification, and MGMT promoter methylation [10–14].

Radiomics extracts high-dimensional quantitative features, such as intensity distributions, spatial relationships, textural heterogeneity, and shape descriptors [15]. The aim of radiomics is to extract quantitative and ideally reproducible information, such as complex patterns that are difficult to recognize [16]. Several studies applied radiomics to predict specific genetic mutations in patients with glioblastoma [17–21]; however, only a few have tried to predict multiple genes simultaneously [21, 22].

There are close associations between IDH mutation and MGMT promoter methylation and between IDH and ATRX mutations [23, 24]. Verhaak et al. classified four gene expression subtypes of glioblastoma and found that EGFR amplification and IDH mutation are major features of classical and proneural glioblastomas, respectively [25]. The Cancer Genome Atlas (TCGA) data analysis showed a tendency towards mutual exclusivity of alterations of components within the mutation pathway [26]. Therefore, a radiomics-based prediction algorithm should integrate the mutual exclusivity among oncogenic pathways and correlations in genetic mutations in glioma [23, 26].

We hypothesized that simultaneous multiple genotype prediction that incorporates relationships among genetic alterations could lead to better performance than multiple independent predictions for each genotype. Therefore, this study aimed to develop a radiomics-based multiple gene prediction model that incorporates mutual information of each genetic alteration in glioblastoma and grade 4 astrocytoma, IDH-mutant.

## Materials and methods

### Patient cohort

We identified patients with newly diagnosed and pathologically confirmed glioblastoma and grade 4 astrocytoma, IDH-mutant at our institution from December 2014 through January 2020. In our electronic medical record system, the diagnosis was glioblastoma, in line with the 2016 WHO classification. Eligible patients met both of the following criteria: (1) had preoperative MRI, including sequences of T1- and T2-weighted image (T1WI and T2WI), FLAIR, and contrast-enhanced T1WI; and (2) had a known specific genetic alteration status, that is, IDH and ATRX mutation, MGMT promoter methylation, and EGFR amplification. This retrospective study was approved by our institutional review board, and the informed consent requirement was waived.

We initially screened 471 patients from medical records. Patients without molecular marker information (n = 20) or preoperative MRI were excluded (n = 11). Patients with the poor image quality of MRI scans or without the full four sequences were also excluded (n = 19). Furthermore, we excluded two patients due to segmentation failure and one due to feature extraction error. Finally, 418 patients were enrolled.

The final study cohort was randomly split into training and test sets (7:3 ratio) while maintaining the proportions of two less-frequent mutations: IDH and ATRX.

### Image processing and radiomic feature extraction

All preoperative MRI scans were performed using a 3 T system MRI with an eight-channel sensitivity-encoding head coil (Achieva or Ingenia, Philips Healthcare). The MRI parameters are described in the Supplementary material. Using T1WI, T2WI, FLAIR, and contrast-enhanced T1WI, a previously described and validated algorithm (HD-GLIO) was used to produce contrast-enhancing tumor (CE) and non-enhancing T2/FLAIR signal abnormality (T2) segmentation masks of the tumors [27, 28]. During this process, image co-registration and brain extraction were also performed. After that, images were resampled to an identical spatial resolution of 1 × 1 × 1 mm. A board-certified neuroradiologist inspected all images and masks to ensure accuracy. These images were subjected to N4 bias correction to remove low-frequency intensity and nonuniformity. After N4 bias correction, Z-score image normalization was done. A total of 660 radiomic features were extracted from the masks on T1WI, T2WI, FLAIR, and contrast-enhanced T1WI using pyradiomics with a bin count of 32 (http://www.radiomics.io/pyradiomics.html) [29].

### Genetic evaluation and molecular subtyping

For IDH and EGFR, mutational and copy number analyses were performed by targeted next-generation sequencing, using the TruSight Tumor 170 panel [23]. Immunohistochemistry was performed using antibodies against ATRX protein. Staining loss in > 50% of tumor cells was considered an ATRX loss case. MGMT promoter methylation was evaluated through a methylation-specific polymerase chain reaction [30].

### Visualization of mutual relations of genetic alterations

**Fig. 1:**
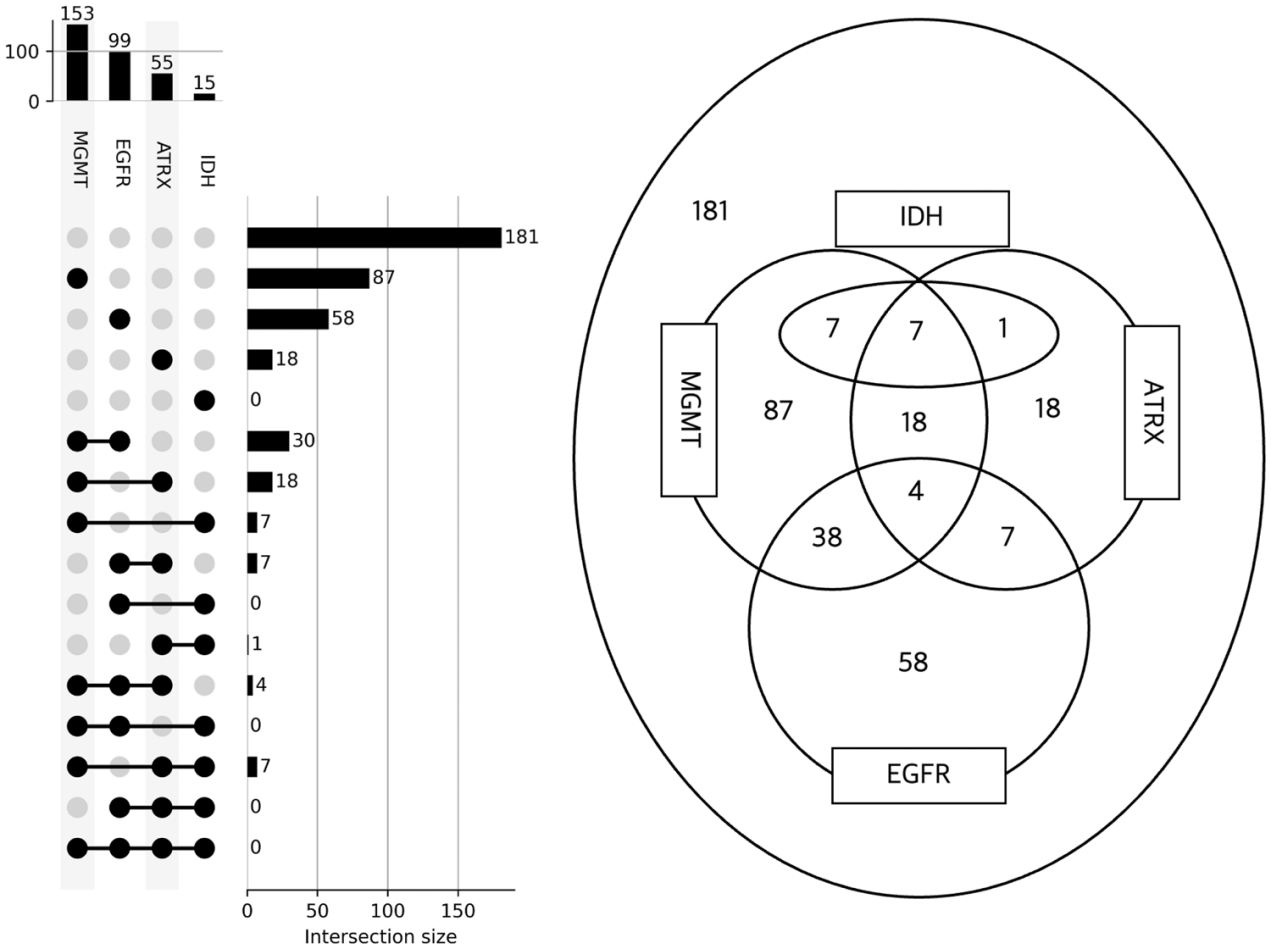
UpSet plot and Venn diagram for the entire cohort

An UpSet plot was drawn based on the frequency table from the entire dataset to examine the relationships between the mutations of IDH, ATRX, MGMT, and EGFR [31]. The UpSet plots the intersections of a set as a matrix (Fig. 1). Each column corresponds to a set, and each row corresponds to one segment in a Venn diagram.

### Multi-label oversampling of training data

Our data had class imbalance, especially for IDH and ATRX. Synthetic Minority Oversampling Technique (SMOTE) is currently one of the most commonly used algorithms to handle this imbalance [32]. We used Multi-Label SMOTE (ML-SMOTE) to mitigate the class imbalance because our task was multi-label classification [33].

### Multi-label classification

**Fig. 2:**
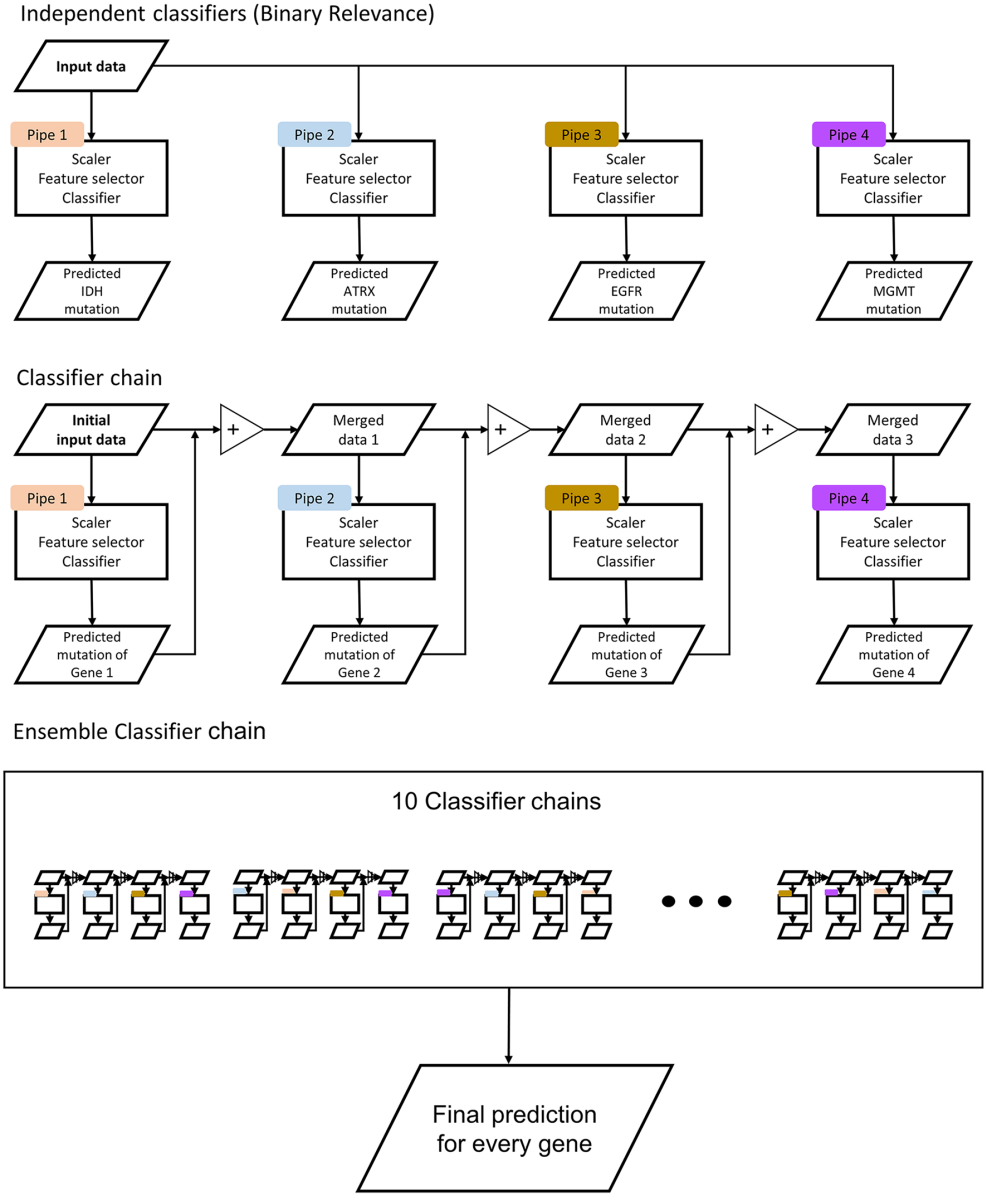
The structure of independent classifiers for the binary relevance and classifier chain models. Ten different classifier chain models were averaged for the ensemble

A common approach to multi-label classification is the binary relevance (BR) method, whereby a multi-label problem is transformed into multiple binary problems, such that a binary model is trained for each label. Classifier chain (CC) is another approach for multi-label classification; it links classifiers along a chain where each classifier deals with a single-label classification [34]. CC is based on the BR method but can take into account label correlations (Fig. 2); therefore, it is often applied in an ensemble framework, whereby multiple chains with different orders of classifiers are ensembled [34]. In this study, we compared the BR and ensemble CC (ECC) methods.

### Model development and validation

For both BR and ECC methods, the basic unit of our models was a pipeline consisting of three components: a *standardizer* using Z-score normalization, a *feature selector* using the least absolute shrinkage and selection operator (LASSO), and a *classifier* using the support vector machine with the linear kernel (Fig. 2). For the BR method, each pipeline for a single label was trained separately. For the ECC method, ten different CCs with random classifier orders were trained, and the mean value of the predicted probabilities was used as the final prediction. Hyperparameter tuning is described in the Supplementary material.

After training the BR and ECC models with the optimized number of input features and C values, the trained models were tested in the test dataset.

### Feature importance for CC

To examine whether the predicted outcomes from earlier classifiers played important roles in the next prediction along a chain and identify radiomics features that were important for predicting mutations in the four genes, we calculated the mean absolute Shapley value for each of the selected input features using the Shapley additive explanations (SHAP) algorithm. For this analysis, the classifier order of IDH > ATRX > MGMT > EGFR was chosen in decreasing order of the test performance. All process up to this point has been done using Python 3 with ScikitLearn library v0.21.2 and the R software (version 3.5.1; R Foundation for Statistical Computing, Vienna, Austria).

## Results

Among 418 enrolled patients (296 males and 122 females; mean age, 60.1 years; Table 1), 3.6%, 13.2%, 36.6%, and 23.7% patients had IDH mutation, ATRX loss, MGMT methylation, and EGFR amplification, respectively. The UpSet plot and Venn diagram of gene alteration information (Fig. 1) showed mutual exclusivity between IDH mutation and EGFR amplification, indicating that a patient with an IDH mutation did not show EGFR amplification and vice versa. Also, every patient with an IDH mutation had MGMT or ATRX gene alteration or both. Therefore, there was no patient with an IDH mutation alone.

**Table 1:** Patient characteristics and gene status in the entire cohort and training and test sets

	Entire cohort (N = 418)	Training set (n = 292)	Test set (n = 126)	*P* value*
Clinical characteristics				
Age (years) ^a^	60.1 ± 13.1	59.6 ± 13.0	61.3 ± 13.0	0.21
Sex				< 0.01
Male	296 (70.8)	224 (76.7)	72 (57.1)	
Female	122 (29.2)	68 (23.3)	54 (42.9)	
Genetic alteration status				
IDH mutation				0.78
Negative	403 (96.4)	282 (96.6)	121 (96)	
Positive	15 (3.6)	10 (3.4)	5 (4.0)	
ATRX loss				0.89
Negative	363 (86.8)	254 (87.0)	109 (86.5)	
Positive	55 (13.2)	38 (13.0)	17 (13.5)	
MGMT promoter methylation				0.18
Negative	265 (63.4)	179 (61.3)	86 (68.3)	
Positive	153 (36.6)	113 (38.7)	40 (31.7)	
EGFR amplification				0.83
Negative	319 (76.3)	223 (76.7)	95 (75.4)	
Positive	99 (23.7)	69 (23.3)	31 (24.6)	

Numbers in parentheses are percentages

*IDH* isocitrate dehydrogenase, *ATRX* alpha-thalassemia/mental retardation syndrome X-linked, *MGMT* O-6-methylguanine-DNA methyltransferase, *EGFR* epidermal growth factor receptor

^*^Calculated using t-test for continuous variables and Chi square test for categorical variables

^a^Data are mean ± standard deviation

Patients were randomly divided into training (n = 292) and test (n = 126) sets (7:3 ratio), and the alteration status of the four genes did not significantly differ between the two sets (Table 1).

After training the BR and ECC models with the optimized number of input features, we evaluated their performance in training and test sets. The micro-averaged AUC for the test set was 0.804 and 0.842 in BR and ECC models, respectively (Supplementary Table 1). The performance (AUC, cutoff value, sensitivity, specificity, positive predictive value, and negative predictive value) for each of the four genes was analyzed (Table 2). ECC model showed higher AUC than BR model for ATRX (0.822 vs. 0.775) and MGMT (0.761 vs. 0.653) predictions. AUCs of IDH prediction were comparable between the two models. The ECC model did not improve the EGFR prediction.

**Table 2:** The performance of models in the test set for each gene

Method	Gene	AUC	TP/FN/TN/FP	Sensitivity (95% CI)*	Specificity (95% CI)^a^
Binary relevance	*IDH*	0.964 (0.922–1)	5/0/107/14	100 (47.8–100)	88.4 (81.3–93.5)
	*ATRX*	0.775 (0.645–0.905)	9/8/104/5	52.9 (27.8–77%)	95.4 (89.6–98.5)
	*MGMT*	0.653 (0.538–0.769)	15/17/73/21	46.9 (29.1–65.3)	77.7 (67.9–85.6)
	*EGFR*	0.753 (0.659–0.847)	18/26/82/0	40.9 (26.3–56.8)	100 (95.6–100)
Ensemble classifier chain	*IDH*	0.967 (0.921–1)	5/0/107/14	100 (47.8–100)	88.4 (81.3–93.5)
	*ATRX*	0.822 (0.687–0.957)	12/5/93/16	70.6 (44–89.7)	85.3 (77.3–91.4)
	*MGMT*	0.761 (0.669–0.853)	21/23/80/2	47.7 (32.5–63.3)	97.6 (91.5–99.7)
	*EGFR*	0.743 (0.642–0.844)	26/6/55/39	81.2 (63.6–92.8)	58.5 (47.9–68.6)

*AUC* area under the receiver operating characteristic curve, *TP* true positive, *FN* false negative, *TN* true negative, *FP* false positive, *CI* confidence interval

^a^Sensitivity, specificity, and their 95% confidence intervals are expressed as percentages

**Fig. 3:**
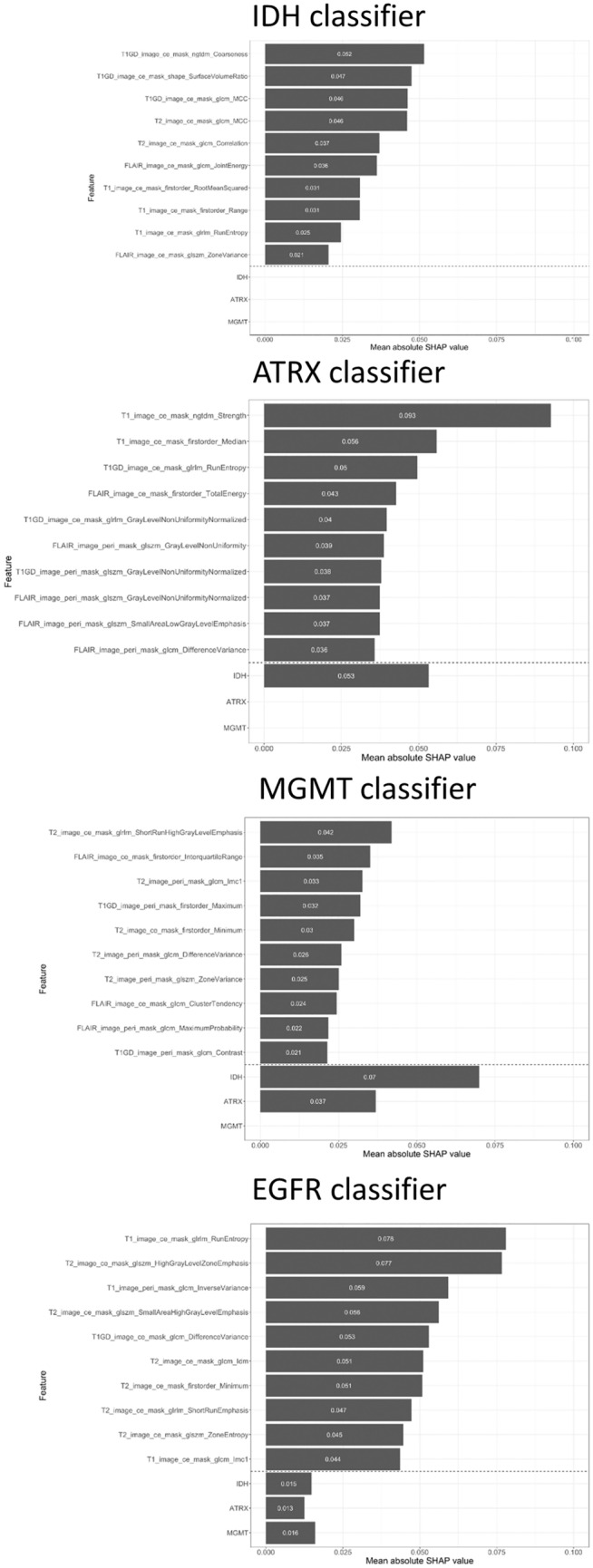
The feature importance presented by mean absolute SHAP value for the prediction of each gene in the classifier chain model from IDH, ATRX, MGMT and EGFR classifier

The mean absolute Shapley value for each of the selected input features was calculated to visualize the feature importance for the ECC model (Fig. 3). The classifier order of IDH > ATRX > MGMT > EGFR was chosen. When the prediction result of IDH was fed to the ATRX classifier, it was the third important feature to predict ATRX status. In the MGMT classifier, prediction results from IDH mutation and ATRX loss were used as important features. The prediction result of IDH was the most important factor to make the MGMT prediction. Finally, IDH, ATRX, and MGMT prediction results were used for the EGFR prediction, and they had relatively similar importance. In the IDH classifier, the top three features with high SHAP values were coarseness (contrast-enhanced T1WI, CE mask), surface volume ratio (contrast-enhanced T1WI, CE mask), and maximal correlation coefficient (contrast-enhanced T1WI and T2WI, CE mask). In the ATRX classifier, the top three features were strength (T1WI, CE mask), median (T1WI, CE mask), and IDH prediction. In the MGMT classifier, the top three features were IDH prediction, short-run high gray-level emphasis (T2WI, CE mask), and ATRX prediction. Finally, for the EGFR prediction, the top three features were run entropy (T1WI, CE mask), high gray-level cone emphasis (T2WI, CE mask), and inverse variance (T1WI, T2 mask).

## Discussion

In this study, we established the radiomics-based simultaneous multiple gene prediction model in glioblastoma and grade 4 astrocytoma, IDH-mutant. Our model predict the gene status of IDH, EGFR, MGMT, and ATRX, using T1WI, T2WI, FLAIR, and contrast-enhanced T1WI sequences. It was a significant investigation because few studies had previously explored the multi-label classification via the radiomics approach in glioblastoma. Furthermore, we improved the multi-label classifier’s performance by applying the ECC model; the final model’s AUC was 0.842 in the test set.

The IDH mutation status is associated with glioma’s prognosis [6, 7]. Several studies have tried predicting the IDH status using MRI features [17, 35–37], and the summary sensitivity and specificity of studies that only included glioblastoma were 90% and 91%, respectively [38]. In our ECC model, coarseness, surface volume ratio, and maximal correlation coefficient from the CE mask were relatively important features; however, a direct comparison of the used radiomics features in the model is difficult because only a few articles mentioned the selected features and their importance in their models, and the classifier development process also varied [17, 39]. Therefore, further investigation of radiomic features’ biological meaning is needed.

A strong association exists between IDH and ATRX mutations [24]. Cancer cells harboring ATRX mutations exhibit chromatin instability and impaired DNA damage response, making them vulnerable to DNA-damaging treatments [40]. An ATRX mutation is usually found in diffuse astrocytomas, generally exclusive to the 1/19q co-deletion [41]. Few studies have investigated the correlation between the imaging features and ATRX loss in glioblastoma [21, 37, 42]. Recently, Ahn et al. used VASARI, which is a system designed to enable consistent description of gliomas using a set of defined visual features and controlled vocabulary, and found that IDH and ATRX mutations clustered according to their shared imaging features [42]. Another study tried to predict the ATRX loss via radiomics features in glioblastoma [21] and enumerated the four most important features. However, we did not find any important features in common. In our model, the probability of IDH mutation predicted by the prior chain was the third most important feature of the ATRX classifier.

MGMT promoter methylation is a favorable prognostic factor in glioblastoma, and patients with MGMT promoter methylation benefit from temozolomide [43, 44]. Previous studies reported around 70% prediction accuracy of MGMT promoter methylation using structural MRI, which is lower than IDH mutation prediction [18, 45, 46]. A study found that ill‐defined tumor borders, lower attenuation coefficients in computed tomography scans, lower fractional anisotropy, and increased apparent diffusion coefficient values are associated with MGMT promoter methylation in a mixed group of WHO grade III and grade IV patients [47]. Ahn et al. showed that biomarkers based on apparent diffusion coefficient and fractional anisotropy parametric maps are poor predictors of MGMT methylation, whereas capillary permeability (i.e., Ktrans) achieved an AUC of 0.76 in whole patient cohort [48]. Korfiatis et al. found that four features (cluster prominence, correlation, inertia, and Haralick correlation) are associated with MGMT methylation [20]. In our study, the AUC for the MGMT methylation prediction was 0.761 (ECC model). According to SHAP value analysis, the prediction result of IDH was the most important feature for the MGMT classifier. IDH mutation increases the overall genomic CpG methylation and is strongly associated with MGMT promoter methylation [49]. In our cohort, 14 of 15 IDH-mutated patients had MGMT promoter methylation; therefore, IDH status could be an important predictor of MGMT status.

EGFR is a type of receptor tyrosine kinase, and its activation results in the activation of multiple downstream signal transduction pathways such as the PI3K/Akt/mTOR pathway [50]. EGFR is altered in approximately 50% of glioblastoma patients. Therefore, detecting the EGFR aberration status could help classify the molecular subtypes and predict treatment response and prognosis in glioblastoma patients. Previous studies reported that EGFR amplification is not related to VASARI image features [51]. Hu et al. built multivariate predictive decision-tree models using radiomics for each glioblastoma driver gene and validated accuracies using leave-one-out cross-validation [22]. They listed several EGFR mutation-related MRI texture features, discrete orthonormal Stockwell transform, gray-level co-occurrence matrix, the standard deviation of raw MRI signal of T2WI, and relative cerebral blood volume’s local binary product. They obtained approximately 75% accuracy using the decision-tree model. However, further study is needed because they did not mention specific features and did not investigate correlations between several other driver genes. In our ECC model, EGFR prediction had the lowest AUC (0.743). The EGFR classifier used the results from IDH, ATRX, and MGMT classifiers; however, the EGFR classifier’s feature importance was not as high as the other classifiers, suggesting that it is relatively difficult to evaluate EGFR status using structural MRI alone. Multiparametric MRI might improve the performance; therefore, further study using multiparametric MRI is needed.

As several genes are highly correlated in glioma, we implemented the multi-label classification. A common approach to multi-label classification is to transform a multi-label problem into one or more single-label problems. The most common approach is the BR method, whereby a multi-label problem is transformed into multiple binary problems. Although BR has the advantage of low computational complexity, its main disadvantage is that it does not take into account the correlations between labels. Another approach is CC, which links classifiers along a chain [34]. CC is based on the BR method but can take into account label correlations (Fig. 2); each model predicts in the order specified by the chain using the predictions of earlier models in the chain. Therefore, CC could overcome the BR method’s disadvantage of ignoring label correlations while inheriting the BR method’s efficiency. CC is often applied in an ensemble framework whereby multiple chains with different orders of classifiers are applied to make the prediction [34, 52]. Our investigation verified the higher performance of simultaneous multiple genotype prediction model (ECC model; considers relationships among genetic alterations) than the performance of independent predictions of each genotype (BR model). Furthermore, we confirmed that each sub-step gene prediction is affected by the previous prediction results in the chained classifier.

Our study has limitations. First, we could not build an independent, external test set from a different institution. Due to the relatively small number of cases with known molecular marker status, it was challenging to enroll enough cases for the external test set. Several open-source databases such as TCGA (https://wiki.cancerimagingarchive.net/display/Public/TCGA-GBM) are available; however, only a few cases had preoperative MRI and information regarding all four genetic alterations. Second, we used ML-SMOTE for training due to disproportionate numbers of patients with IDH and ATRX mutations, which may have impacted our model’s performance. In the presence of class imbalance, machine learning algorithms are biased towards predicting the majority class as they do not have enough data to learn the patterns present in the minority class. The SMOTE algorithm takes the samples belonging to the minority class, chooses a random sample among the nearest neighbors of each, and synthesizes a new sample belonging to the same minority class [32]. However, in multi-label classification, the associations between the labels must be considered when creating new label sets. Thus, we used ML-SMOTE [33], which synthesizes new samples (i.e., radiomics features) belonging to multiple minority labels as well as a set of labels (i.e., the presence or absence of IDH, ATRX, MGMT, and EGFR mutations), taking into consideration the label correlation information. Finally, our chained classifier model was based only on four major genes. There may be more interacting genes that we could not include, and these four genes are possibly interacting indirectly. We could have better estimated the complex interactions among many genetic mutations if we had more information regarding various genetic alterations.

In conclusion, we built a radiomics-based multiple gene prediction chained model that incorporates mutually exclusive information of each genetic alteration in glioblastoma and grade 4 astrocytoma, IDH-mutant. The chained model used the prior classifiers’ prediction probabilities and performed better than the simple bundle of classifiers.

## Supplementary Information

Below is the link to the electronic supplementary material.

Supplementary file1 (DOCX 490 kb)

## Data Availability

The datasets generated during and/or analyzed during the current study are available from the corresponding author on reasonable request.

## Code availability

The code generated during the current study are available from the corresponding author on reasonable request.

## References

[1] Stupp R, Hegi ME, Mason WP (2009). Effects of radiotherapy with concomitant and adjuvant temozolomide versus radiotherapy alone on survival in glioblastoma in a randomised phase III study: 5-year analysis of the EORTC-NCIC trial. Lancet Oncol.

[2] Touat M, Idbaih A, Sanson M, Ligon KL (2017). Glioblastoma targeted therapy: updated approaches from recent biological insights. Ann Oncol.

[3] Reardon DA, Wen PY, Mellinghoff IK (2014). Targeted molecular therapies against epidermal growth factor receptor: past experiences and challenges. Neuro Oncol.

[4] Rohle D, Popovici-Muller J, Palaskas N (2013). An inhibitor of mutant IDH1 delays growth and promotes differentiation of glioma cells. Science.

[5] Gallego O, Cuatrecasas M, Benavides M (2014). Efficacy of erlotinib in patients with relapsed gliobastoma multiforme who expressed EGFRVIII and PTEN determined by immunohistochemistry. J Neurooncol.

[6] Beiko J, Suki D, Hess KR (2014). IDH1 mutant malignant astrocytomas are more amenable to surgical resection and have a survival benefit associated with maximal surgical resection. Neuro Oncol.

[7] Sanson M, Marie Y, Paris S (2009). Isocitrate dehydrogenase 1 codon 132 mutation is an important prognostic biomarker in gliomas. J Clin Oncol.

[8] Louis DN, Perry A, Reifenberger G, Von Deimling A, Figarella-Branger D, Cavenee WK, Ohgaki H, Wiestler OD, Kleihues P, Ellison DW (2016). The 2016 World Health Organization classification of tumors of the central nervous system: a summary. Acta Neuropathol.

[9] Louis DN, Perry A, Wesseling P, Brat DJ, Cree IA, Figarella-Branger D, Hawkins C, Ng H, Pfister SM, Reifenberger G (2021). The 2021 WHO classification of tumors of the central nervous system: a summary. Neuro Oncol.

[10] Carrillo JA, Lai A, Nghiemphu PL (2012). Relationship between tumor enhancement, edema, IDH1 mutational status, MGMT promoter methylation, and survival in glioblastoma. AJNR Am J Neuroradiol.

[11] Ellingson BM (2015). Radiogenomics and imaging phenotypes in glioblastoma: novel observations and correlation with molecular characteristics. Curr Neurol Neurosci Rep.

[12] Zhang B, Chang K, Ramkissoon S, Tanguturi S, Bi WL, Reardon DA, Ligon KL, Alexander BM, Wen PY, Huang RY (2017). Multimodal MRI features predict isocitrate dehydrogenase genotype in high-grade gliomas. Neuro Oncol.

[13] Gutman DA, Dunn WD, Grossmann P, Cooper LA, Holder CA, Ligon KL, Alexander BM, Aerts HJ (2015). Somatic mutations associated with MRI-derived volumetric features in glioblastoma. Neuroradiology.

[14] Park CJ, Han K, Kim H, Ahn SS, Choi D, Park YW, Chang JH, Kim SH, Cha S, Lee SK (2021). MRI features may predict molecular features of glioblastoma in isocitrate dehydrogenase wild-type lower-grade gliomas. AJNR Am J Neuroradiol.

[15] Aerts HJ, Velazquez ER, Leijenaar RT (2014). Decoding tumour phenotype by noninvasive imaging using a quantitative radiomics approach. Nat Commun.

[16] Gillies RJ, Kinahan PE, Hricak H (2016). Radiomics: images are more than pictures, they are data. Radiology.

[17] Su X, Sun H, Chen N, Roberts N, Yang X, Wang W, Li J, Huang X, Gong Q, Yue Q (2020). A radiomics-clinical nomogram for preoperative prediction of IDH1 mutation in primary glioblastoma multiforme. Clin Radiol.

[18] Sasaki T, Kinoshita M, Fujita K (2019). Radiomics and MGMT promoter methylation for prognostication of newly diagnosed glioblastoma. Sci Rep.

[19] Crisi G, Filice S (2020). Predicting MGMT promoter methylation of glioblastoma from dynamic susceptibility contrast perfusion: a radiomic approach. J Neuroimaging.

[20] Korfiatis P, Kline TL, Coufalova L, Lachance DH, Parney IF, Carter RE, Buckner JC, Erickson BJ (2016). MRI texture features as biomarkers to predict MGMT methylation status in glioblastomas. Med Phys.

[21] Calabrese E, Villanueva-Meyer JE, Cha S (2020). A fully automated artificial intelligence method for non-invasive, imaging-based identification of genetic alterations in glioblastomas. Sci Rep.

[22] Hu LS, Ning S, Eschbacher JM (2017). Radiogenomics to characterize regional genetic heterogeneity in glioblastoma. Neuro Oncol.

[23] Na K, Kim HS, Shim HS, Chang JH, Kang SG, Kim SH (2019). Targeted next-generation sequencing panel (TruSight Tumor 170) in diffuse glioma: a single institutional experience of 135 cases. J Neurooncol.

[24] Kannan K, Inagaki A, Silber J, Gorovets D, Zhang J, Kastenhuber ER, Heguy A, Petrini JH, Chan TA, Huse JT (2012). Whole-exome sequencing identifies ATRX mutation as a key molecular determinant in lower-grade glioma. Oncotarget.

[25] Verhaak RG, Hoadley KA, Purdom E (2010). Integrated genomic analysis identifies clinically relevant subtypes of glioblastoma characterized by abnormalities in PDGFRA, IDH1, EGFR, and NF1. Cancer Cell.

[26] Cancer Genome Atlas Research N (2008). Comprehensive genomic characterization defines human glioblastoma genes and core pathways. Nature.

[27] Kickingereder P, Isensee F, Tursunova I (2019). Automated quantitative tumour response assessment of MRI in neuro-oncology with artificial neural networks: a multicentre, retrospective study. Lancet Oncol.

[28] Isensee F, Jaeger PF, Kohl SAA, Petersen J, Maier-Hein KH (2021). nnU-Net: a self-configuring method for deep learning-based biomedical image segmentation. Nat Methods.

[29] van Griethuysen JJM, Fedorov A, Parmar C, Hosny A, Aucoin N, Narayan V, Beets-Tan RGH, Fillion-Robin JC, Pieper S, Aerts H (2017). Computational radiomics system to decode the radiographic phenotype. Cancer Res.

[30] Vlassenbroeck I, Califice S, Diserens AC (2008). Validation of real-time methylation-specific PCR to determine O6-methylguanine-DNA methyltransferase gene promoter methylation in glioma. J Mol Diagn.

[31] Lex A, Gehlenborg N, Strobelt H, Vuillemot R, Pfister H (2014). UpSet: visualization of intersecting sets. IEEE Trans Vis Comput Graph.

[32] Chawla NV, Bowyer KW, Hall LO, Kegelmeyer WP (2002). SMOTE: synthetic minority over-sampling technique. J Artif Intell Res.

[33] Charte F, Rivera AJ, del Jesus MJ, Herrera F (2015). MLSMOTE: approaching imbalanced multilabel learning through synthetic instance generation. Knowl-Based Syst.

[34] Simon GJ, Kumar V, Li PW (2011) A simple statistical model and association rule filtering for classification. In: Proceedings of the 17th ACM SIGKDD international conference on Knowledge discovery and data mining, pp 823–831

[35] Lee MH, Kim J, Kim ST, Shin HM, You HJ, Choi JW, Seol HJ, Nam DH, Lee JI, Kong DS (2019). Prediction of IDH1 mutation status in glioblastoma using machine learning technique based on quantitative radiomic data. World Neurosurg.

[36] Hsieh KL, Chen CY, Lo CM (2017). Radiomic model for predicting mutations in the isocitrate dehydrogenase gene in glioblastomas. Oncotarget.

[37] Hong EK, Choi SH, Shin DJ (2018). Radiogenomics correlation between MR imaging features and major genetic profiles in glioblastoma. Eur Radiol.

[38] Suh CH, Kim HS, Jung SC, Choi CG, Kim SJ (2019). Imaging prediction of isocitrate dehydrogenase (IDH) mutation in patients with glioma: a systemic review and meta-analysis. Eur Radiol.

[39] Choi Y, Nam Y, Lee YS, Kim J, Ahn KJ, Jang J, Shin NY, Kim BS, Jeon SS (2020). IDH1 mutation prediction using MR-based radiomics in glioblastoma: comparison between manual and fully automated deep learning-based approach of tumor segmentation. Eur J Radiol.

[40] Koschmann C, Calinescu AA, Nunez FJ (2016). ATRX loss promotes tumor growth and impairs nonhomologous end joining DNA repair in glioma. Sci Transl Med.

[41] Reuss DE, Sahm F, Schrimpf D (2015). ATRX and IDH1-R132H immunohistochemistry with subsequent copy number analysis and IDH sequencing as a basis for an "integrated" diagnostic approach for adult astrocytoma, oligodendroglioma and glioblastoma. Acta Neuropathol.

[42] Ahn SS, An C, Park YW, Han K, Chang JH, Kim SH, Lee S-K, Cha S (2021). Identification of magnetic resonance imaging features for the prediction of molecular profiles of newly diagnosed glioblastoma. J Neuro-Oncol.

[43] Chinot OL, Barrie M, Fuentes S (2007). Correlation between O6-methylguanine-DNA methyltransferase and survival in inoperable newly diagnosed glioblastoma patients treated with neoadjuvant temozolomide. J Clin Oncol.

[44] Hegi ME, Diserens AC, Gorlia T (2005). MGMT gene silencing and benefit from temozolomide in glioblastoma. N Engl J Med.

[45] Drabycz S, Roldan G, de Robles P, Adler D, McIntyre JB, Magliocco AM, Cairncross JG, Mitchell JR (2010). An analysis of image texture, tumor location, and MGMT promoter methylation in glioblastoma using magnetic resonance imaging. Neuroimage.

[46] Kanas VG, Zacharaki EI, Thomas GA, Zinn PO, Megalooikonomou V, Colen RR (2017). Learning MRI-based classification models for MGMT methylation status prediction in glioblastoma. Comput Methods Programs Biomed.

[47] Moon WJ, Choi JW, Roh HG, Lim SD, Koh YC (2012). Imaging parameters of high grade gliomas in relation to the MGMT promoter methylation status: the CT, diffusion tensor imaging, and perfusion MR imaging. Neuroradiology.

[48] Ahn SS, Shin NY, Chang JH, Kim SH, Kim EH, Kim DW, Lee SK (2014). Prediction of methylguanine methyltransferase promoter methylation in glioblastoma using dynamic contrast-enhanced magnetic resonance and diffusion tensor imaging. J Neurosurg.

[49] Horbinski C, McCortney K, Stupp R (2021). MGMT promoter methylation is associated with patient age and 1p/19q status in IDH-mutant gliomas. Neuro-Oncol.

[50] Mao H, Lebrun DG, Yang J, Zhu VF, Li M (2012). Deregulated signaling pathways in glioblastoma multiforme: molecular mechanisms and therapeutic targets. Cancer Invest.

[51] Jain R, Poisson LM, Gutman D, Scarpace L, Hwang SN, Holder CA, Wintermark M, Rao A, Colen RR, Kirby J (2014). Outcome prediction in patients with glioblastoma by using imaging, clinical, and genomic biomarkers: focus on the nonenhancing component of the tumor. Radiology.

[52] Read J, Pfahringer B, Holmes G, Frank E (2011). Classifier chains for multi-label classification. Mach Learn.

