# Supplementary material for: Radiomics-based prediction of multiple gene alteration incorporating mutual genetic information in glioblastoma and grade 4 astrocytoma, IDH-mutant
Source: J Neurooncol. 2021 Oct 14;155(3):267–76. doi: 10.1007/s11060-021-03870-z (PMC8651601; doi:10.1007/s11060-021-03870-z)
Supplement: Supplementary file 1 — Supplementary file1 (DOCX 490 kb) [file 11060_2021_3870_MOESM1_ESM.docx]

**Radiomics-based prediction of multiple gene alterations incorporating mutual genetic information in glioblastoma and grade 4 astrocytoma, IDH-mutant**

**Supplementary Material**

**MRI parameters**

The preoperative MRI protocol included axial 3D T2-weighted imaging (WI) fast spin echo (TR/TE = 3000/102 ms), 3D fluid-attenuated inversion recovery (TR/TE = 10,000/148 ms; inversion time = 2200 ms), and contrast-enhanced 3D spoiled gradient echo T1WI (T1C) (TR/TE=34/8 ms; in-plane field of view = 260 mm; section thickness = 1.5 mm; intersection gap = 0 mm; matrix = 256×256). T1C images were obtained after administration of a gadolinium-based contrast agent (Gadovist, Bayer, Toronto, ON, Canada) at a dose of 0.1 mL/kg.

**Hyperparameter tuning**

For hyperparameter tuning, we performed five-fold cross-validation in the training dataset using the micro-averaged area under the curve (AUC) as an evaluation metric. Hyperparameters that we tuned to find optimal models were the number of input features and the C value for the support vector machine. The same hyperparameters were applied to the unit pipelines within a binary relevance (BR) or ensemble classifier chain (ECC) model. To avoid overfitting, we first tried to find an optimal number of features using early stopping before determining the optimal C value. For each number of features (from 1 to 100), ten mean cross-validated AUCs were obtained using different C values (0.001, 0.005, 0.01, 0.05, 0.1, 0.5, 1, 2, 5, and 10) and averaged. If the average mean AUC did not improve even after incrementing the number of features ten times, the search was stopped, and the number of features that showed the best performance was determined to be optimal. Next, with the number of features set to the optimized value, the C value showing the lowest standard deviation of cross-validated AUCs was selected as the optimal value. Consequently, each model’s optimal number of features was determined: 47 features for the BR model and 61 features for the ECC model.


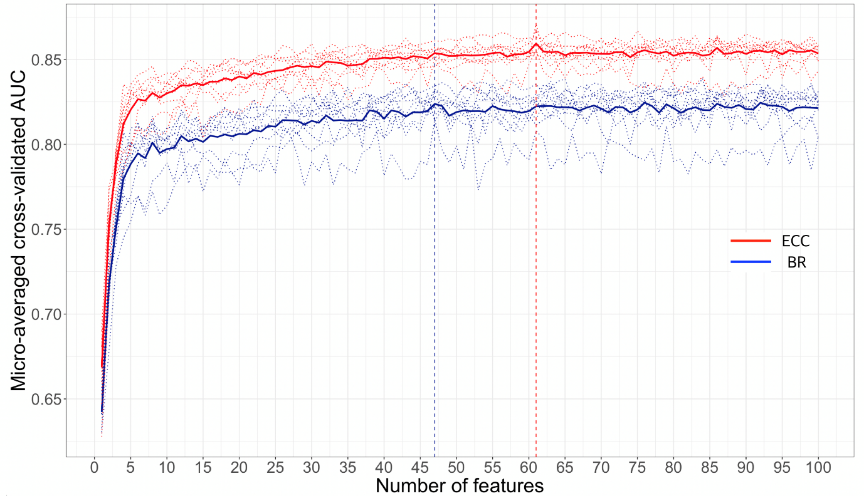


Fig. S1 Hyperparameter tuning. Micro-averaged area under the receiver operating characteristic curve (AUC) was changed as the number of features increased. The vertical dashed line is the chosen point for the optimal number of features in each model. Thin dashed line: AUC for each cross validation. Thick solid line: Micro-average of AUC.

**Supplementary Table 1** The performance of models in the training and test sets

| Method | Cross-Validation Results | | | Test Performance |
| --- | --- | --- | --- | --- |
|  | Number of Features | C Value | Micro-averaged AUC (Standard Deviation) | Micro-averaged AUC  (95% CI) |
| Binary relevance | 47 | 0.1 | 0.821 (0.011) | 0.804 (0.757–0.85) |
| Ensemble classifier chain | 61 | 0.01 | 0.871 (0.013) | 0.842 (0.8–0.883) |
| AUC, area under the receiver operating characteristic curve; CI, confidence interval | | | | |
